# Analysis of Volatile Organic Compounds Liberated and Metabolised by Human Umbilical Vein Endothelial Cells (HUVEC) In Vitro

**DOI:** 10.1007/s12013-014-0201-4

**Published:** 2014-08-15

**Authors:** Paweł Mochalski, Markus Theurl, Andreas Sponring, Karl Unterkofler, Rudolf Kirchmair, Anton Amann

**Affiliations:** 1 Breath Research Institute, University of Innsbruck, Rathausplatz 4, 6850 Dornbirn, Austria; 2 Institute of Nuclear Physics PAN, Radzikowskiego 152, 31342 Kraków, Poland; 3 Univ.-Clinic for Internal Medicine III, Innsbruck Medical University, Anichstr, 35, 6020 Innsbruck, Austria; 4 Univ.-Clinic for Anesthesia, Innsbruck Medical University, Anichstr, 35, 6020 Innsbruck, Austria

**Keywords:** HUVEC, Volatile organic compounds, GC–MS, Needle trap, Uptake, Release, Aldehydes

## Abstract

Gas chromatography with mass spectrometric detection combined with head-space needle trap extraction as the pre-concentration technique was applied to identify and quantify volatile organic compounds released or metabolised by human umbilical vein endothelial cells. Amongst the consumed species there were eight aldehydes (2-methyl 2-propenal, 2-methyl propanal, 2-methyl butanal, 3-methyl butanal, *n*-hexanal, benzaldehyde, *n*-octanal and *n*-nonanal) and *n*-butyl acetate. Further eight compounds (ethyl acetate, ethyl propanoate, ethyl butyrate, 3-heptanone, 2-octanone, 2-nonanone, 2-methyl-5-(methylthio)-furan and toluene) were found to be emitted by the cells under study. Possible metabolic pathways leading to the uptake and release of these compounds by HUVEC are proposed and discussed. The uptake of aldehydes by endothelial cells questions the reliability of species from this chemical class as breath or blood markers of disease processes in human organism. The analysis of volatiles released or emitted by cell lines is shown to have a potential for the identification and assessment of enzymes activities and expression.

## Introduction

There is considerable evidence that volatile organic compounds (VOCs) released by human organism mirror normal physiological processes as well as pathological disorders and have, thereby, a great potential for medical diagnosis and therapy [1–4]. Breath analysis holds in this context a distinguished status as it is non-invasive and some breath constituents have already been linked to various disease processes [5–10]. The main obstacle limiting the clinical application of breath tests is the insufficient understanding of the origin and metabolic fate of breath markers. In vitro studies involving pathogenic microorganisms (e.g. bacteria, fungi) or human cells (both normal and cancerogenous) are, within this framework, an invaluable tool capable of revealing the biochemical pathways of breath biomarkers production or metabolism. For instance, over the last few years, a substantial progress was made to pinpoint volatiles emitted or consumed by cancer cells [11–16], bacteria [17, 18], or fungi [19].

In the current study, human umbilical vein endothelial cells (HUVEC) were investigated. These cells are isolated from the vein of the umbilical cord and are commonly used for physiological and pharmacological investigations [20–22]. In breath gas analysis, endothelial cells play a crucial role as they form the interior surface of the vascular system—a trunk line transporting volatile markers from distant parts of the body to lungs. Consequently, the uptake and release of VOCs by these cells can considerably modify the profile of blood and breath VOCs and, thereby, distort the information they provide. Hence, the main goal of this work was to identify and quantify volatile organic compounds being emitted or metabolised by human umbilical vein endothelial cells. Their determination relied on gas chromatography with mass spectrometric detection (GC–MS) and head-space needle trap extraction (HS-NTE) as the pre-concentration method.

## Materials and Methods

### Chemicals and Standards

Calibration mixtures were prepared from high-purity liquid substances. The majority of them were purchased from Sigma-Aldrich (Austria): 2-methyl 2-propenal (95 %), 2-methyl propanal (99.5 %), 3-methyl butanal (97 %), *n*-hexanal (98 %), *n*-octanal (99 %), ethyl butyrate (99 %), toluene (99.8 %) and ethyl acetate (99.9 %). Moreover, *n*-butyl acetate (99.7 %), benzaldehyde (99 %), *n*-nonanal (95 %), 2-octanone (99.5 %) and 2-methyl butanal (99 %) were obtained from Fluka (Switzerland), whereas ethyl propanoate (97 %) was provided by SAFC (USA). 3-heptanone (98 %) was purchased from Alfa Aesar (USA), 2-methyl-5-(methylthio) furan (99 %) from Chemos (Germany) and 2-nonanone (98 %) was purchased from Merck Schuchardt (Germany).

Gaseous humid calibration mixtures were prepared using a GasLab calibration mixtures generator (Breitfuss Messtechnik, Germany). The GasLab consists of an integrated zero air generator, a 2-stage dynamic injection module, for evaporating a liquid and diluting it with zero air, and a humidification module enabling the preparation of gas mixtures at pre-defined humidity levels. When using pure liquid substances, GasLab produces a flow of up to 10 L/min of complex trace gas mixtures in dry or humidified zero air containing 10 ppb–100 ppm of each solute. Since in this study the observed levels of compounds of interest were much lower, pure substances were additionally diluted (1:1,000–1:2,000) with distilled water or methanol prior to the evaporation. Effectively, humid gas mixtures (100 % RH at 37 °C) with volume fractions ranging from approximately 0.04 to 150 ppb were used for the purpose of calibration and validation.

### Cell Cultivation

**Fig. 1:**
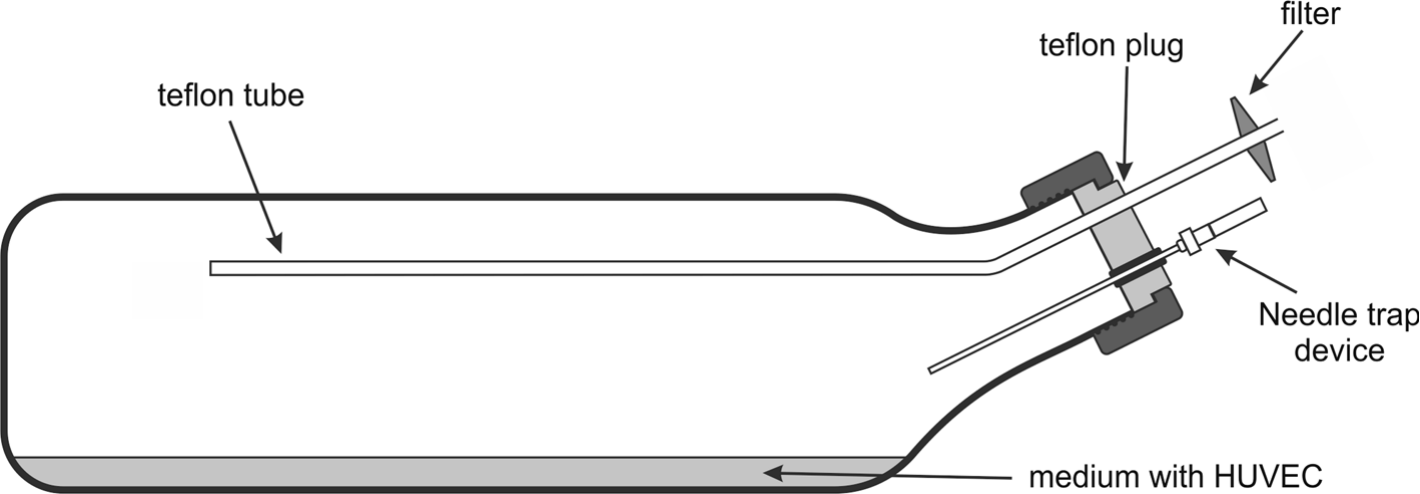
Cultivation/measurement bottle

For the experiments human umbilical vein endothelial cells (HUVEC) passage 5 (P5) from pooled donors were used (PromoCell, C-12203). After building up a confluent monolayer (cell density 80–90 %) in a 75 cm^2^ cell culture bottle HUVEC were split 1:3 to cultivation glass bottles coated with 0.2 % gelatin solution (Sigma, G1393). The cultivation/measurement bottles had diameters of 21 cm × 5.5 cm × 11.5 cm (1,000 mL nominal volume, bottom area of approximately 240 cm^2^) and are shown in Fig. 1. The bottles were closed with a special Teflon plug equipped with a rubber septum enabling the insertion of the needle trap devices into the head-space of the culture and the Teflon tube being the inlet of the zero air. The inner end of the Teflon tube protruded 15–17 cm from the plug into the head-space volume, whereas the outer end was equipped with a sterile filter. The cell culture medium (EBM-2, CC-3156, supplemented with EGM-2 single quotes, CC-4147; both Clonetics) was changed every other day. After building up a confluent monolayer in the glass bottles, cells were washed three times with PBS (PAA, H15-002) and cultured in 30 mL medium. A glass bottle coated with gelatin solution (no cells) and filled with medium was used as background control. HUVEC were incubated for 24–30 h in a humidified atmosphere (37 °C and 5 % CO_2_) and consequently processed for the GC–MS analyses of the head-space composition. For the 24 to 30 h of cultivation, the bottles were tightly closed to boost the accumulation of species released by the cells and to block the gas exchange with the ambient air. Cell viability counts (trypan blue exclusion method) were performed immediately after the measurements. In total, 7 experiments (each involving 1 cell culture and 1 control) were performed.

### Head-Space Sampling Procedure and Chromatographic Analysis

Head-space volatile organic compounds were pre-concentrated using three-bed side-hole 23-gauge stainless steel needle trap devices (NTD) (PAS Technology, Germany) [23, 24]. All needles were Silcosteel-treated and their sorbent beds consisted of 1 cm of Tenax TA (80/100 mesh), 1 cm of Carbopack X (60/80 mesh) and 1 cm of Carboxen 1,000 (60/80 mesh). Prior to the first use, all NTDs were pre-conditioned at 290 °C by flushing them with high-purity nitrogen (6.0–99.9999 %) for 4 h. Their re-conditioning was performed before each sampling, however, with shorter flushing times of 10 min. Since NTDs exhibited relatively huge disparities with respect to the extraction efficiency (deviations of up to 70 %, even when originating from the same production lot), the NTDs used during experiments were pre-selected according to the condition that their inter-needle variability should be below 10 %.

The sampling was performed dynamically by inserting the NTD through a rubber septum into the head-space of the bottle and drawing 200 mL of head-space gas at a steady flow rate of 10 mL/min at 37 °C. These conditions were achieved with the help of a membrane pump (Vacuubrand, Germany) and a mass flow controller (RED-Y, Burde Co. GmbH, Austria). Consequently, no transfer line was present between the sampled head-space and a needle trap. To maintain the constant pressure in the bottle during sampling, high-purity zero air was continuously introduced into it via the Teflon tube (see Fig. 1) at a flow equal to the sampling flow. Immediately after an extraction, the NTD was manually introduced into the inlet of the gas chromatograph where the compounds of interest were thermally desorbed at 290 °C in a splitless mode (1 min).

Chromatographic analyses were performed using an Agilent 7890A/5975C GC–MS system (Agilent, USA). During desorption, the split/splitless inlet operated in the splitless mode (1 min), followed by a split mode at ratio 1:20. The analytes were separated using a PoraBond Q column (25 m x 0.32 mm, film thickness 5 μm, styrene–divinylbenzene copolymer phase, Varian, USA) working under a constant flow of helium (1.5 mL/min). The column temperature programme was as follows: 40 °C for 5 min, increase to 260 °C at a rate of 7 °C/min, followed by a constant temperature phase at 260 °C for 6 min. The mass spectrometer worked in a SCAN mode with an m/z range set from 20 to 200. The quadrupole, ion source and transfer line temperatures were kept at 150, 230 and 280 °C, respectively.

The identification of compounds was performed in two steps. First, the peak spectrum was checked against the NIST mass spectral library (NIST/EPA/NIH mass spectral library version 2.0f). Next, the NIST tentative identifications were validated by collating the respective retention times with the list of retention times obtained on the basis of analyses of standard mixtures. Peak integration was based on extracted ion chromatograms. The retention times of the investigated compounds for the applied chromatographic parameters as well as the quantifier ions used for the integration are presented in Table 1.

**Table 1:** Retention times *R*_t_ (min), quantifier ions, LODs (ppb), RSDs (%), coefficients of variation (*R* ^2^) and linear ranges (ppb) of compounds under study

VOC	CAS	*R*_t_ (min)	Quantifier ion	LOD (ppb)	RSD (%)	*R* ^2^	Linear range (ppb)
2-Propenal, 2-methyl-	78-85-3	18.99	70	0.03	8	0.993	0.1–12
Propanal, 2-methyl-	78-84-2	19.27	72	0.3	9	0.977	0.9–150
Ethyl acetate	141-78-6	21.00	43	0.12	5.5	0.987	0.36–20
Butanal, 3-methyl-	590-86-3	23.36	44	0.14	9	0.978	0.4–170
Butanal, 2-methyl-	96-17-3	23.42	Not quantified, RT confirmed				
Ethyl propanoate	105-37-3	24.64	57	0.04	9	0.998	0.12–1.5
Toluene	108-88-3	26.21	91	0.1	6	0.999	0.3–20
*n*-Hexanal	66-25-1	27.73	56	0.2	9	0.994	0.6–15
Ethyl butyrate	105-54-4	27.93	71	0.02	9	0.996	0.06–1
*n*-Butyl acetate	123-86-4	28.18	56	0.04	10	0.997	0.12–8
3-Heptanone	106-35-4	30.51	85	0.03	2.5	0.997	0.09–7
Benzaldehyde	100-52-7	30.87	106	0.05	12	0.998	0.15–12
Furan, 2-methyl-5-(methylthio)-	13678-59-6	31.00	128	0.03	7	0.988	0.09–4
2-Octanone	111-13-7	33.56	58	0.05	9	0.991	0.15–5.5
*n*-Octanal	124-13-0	33.76	84	0.1	10	0.993	0.3–13
2-Nonanone	821-55-6	36.19	58	0.07	11	0.974	0.21–5.7
*n*-Nonanal	124-19-6	36.41	57	0.4	12	0.930	1.2–12

Compounds are ordered with respect to increasing retention time

## Results and Discussion

### Validation Parameters

Limits of detection (LODs) were calculated using the mean value of the blank responses and their standard deviations obtained on the basis of 10 blank measurements [25]. The LOD values ranged from 0.02 ppb for ethyl butyrate to 0.3 ppb for 2-methyl propanal, see Table 1. The relative standard deviations (RSDs) were calculated on the basis of five consecutive analyses of humid standard mixtures. The calculated RSDs varied from 2.5 to 12 % and were recognised as adequate for the aim of this study. The system response was found to be linear within the investigated concentration ranges with the coefficients of variation ranging from 0.930 to 0.999, as shown in Table 1.

### HUVEC Cultures

The total number of HUVEC and their viabilities after 24–30 h incubation in the closed measurement bottles are shown in Table 2. The total number of cells fell within the range of 3.3 mio and 45.5 mio (mean 15.8 mio), whereas the viability varied from 81.4 to 99.8 % (mean 92.7 %). Consequently, the applied experimental protocol did not affect relevantly the cells’ viability, and it can be assumed that the release and uptake of the head-space VOCs mirror their metabolism.

**Table 2:** Total number of cells, number of living cells and viability at the end of the cultivation period

Culture	Total number of cells (mio)	Number of living cells (mio)	Viability (%)
1	13.6	13.2	97.4
2	45.5	42.8	93.9
3	3.3	2.7	83.7
4	5.3	4.3	81.4
5	12.8	12.1	94.8
6	14	13.7	97.7
7	15.9	15.9	99.8
Mean	15.8	14.9	92.7

### Uptake of VOCs by HUVEC

A total of nine species were found to be consumed by the HUVEC (Wilcoxon signed-rank test, *p* < .05). Their incidences and concentration ranges in the head-space of cell cultures and controls are given in Table 3. The predominant chemical class in this group were aldehydes with eight compounds (2-methyl 2-propenal, 2-methyl propanal, 2-methyl butanal, 3-methyl butanal, *n*-hexanal, benzaldehyde, *n*-octanal and *n*-nonanal). Apart from aldehydes there was one ester, *n*-butyl acetate. In the case of 2-methyl butanal, proper integration and quantification was not possible due to the poor separation from 3-methyl butanal and the absence of unique ion that could be used for these purposes. Aliphatic and saturated aldehydes seemed to be more preferred substrates for HUVEC than unsaturated or aromatic ones. For instance, the levels of *n*-hexanal and 3-methyl butanal were reduced by approximately 90 % after the 1-day-long incubation, whereas the concentrations of 2-methyl 2-propenal and benzaldehyde dropped only by 40 and 60 %, respectively.

**Table 3:** Detection (*n*_d_) and quantification (*n*_q_) incidences and ranges (means) of VOCs under study in the head-space of medium and cell cultures

VOC	CAS	Cell cultures		Medium	
		Incidence *n*_d_ (*n*_q_)	Range (mean) (ppb)	Incidence *n*_d_ (*n*_q_)	Range (mean) (ppb)
Uptake					
2-Propenal, 2-methyl-	78-85-3	7(5)	0.67–3.1(1.5)	7(7)	0.9–4.1(2.4)
Propanal, 2-methyl-	78-84-2	7(7)	1.5–16(5.6)	7(7)	16–125 (56)
Butanal, 3-methyl-	590-86-3	6(6)	1.2–17.2(5.3)	7(7)	1.7–95.5(44)
*n*-Hexanal	66-25-1	7(6)	0.7–3(1.5)	7(7)	5.8–15.5(9.8)
*n*-Butyl acetate	123-86-4	7(4)	0.13–0.58(0.38)	7(7)	0.15–0.88(0.52)
Benzaldehyde	100-52-7	7(7)	0.17–1.1(0.48)	7(7)	0.63–2.53(1.2)
*n*-Octanal	124-13-0	4(0)	<LOQ	7(7)	0.32–3.34(0.98)
*n*-Nonanal	124-19-6	1(0)	<LOQ	7(6)	1.8–2.2(2.0)
Release					
Ethyl acetate	141-78-6	7(7)	3.7–16.2(10.1)	7(7)	0.5–3.2(1.8)
Ethyl propanoate	105-37-3	7(7)	0.14–0.90(0.49)	6(0)	<LOQ
Toluene	108-88-3	7(7)	1.8–18.6(7.8)	7(7)	1.2–5.9(3.6)
Ethyl butyrate	105-54-4	7(6)	0.07–0.22(0.16)	1(0)	<LOQ
3-Heptanone	106-35-4	7(7)	0.3–1.6(1.0)	7(7)	0.1–0.79(0.45)
Furan, 2-methyl-5-(methylthio)-	13678-59-6	7(7)	0.11–0.36(0.25)	0(0)	<LOD
2-Octanone	111-13-7	7(6)	0.18–0.39(0.28)	7(1)	0.16
2-Nonanone	821-55-6	7(6)	0.25–0.50(0.37)	6(0)	<LOQ

A potential pathway leading to the uptake of aldehydes by HUVEC involves aldehyde dehydrogenases (ALDHs). ALDHs irreversibly oxidise a wide spectrum of endogenous and exogenous aldehydes to their corresponding carboxylic acids [26, 27]. Although ALDHs in endothelial cells are rather poorly expressed, their activity has been evidenced in the literature [28, 29]. Moreover, the observed differences in the uptake of different types of aldehydes are consistent with the reported specificity of human ALDHs towards species from this chemical class [26]. Alternatively, aldehydes can be reduced to alcohols by alcohol dehydrogenases (ADHs). ADHs were found to be abundant in human blood vessels; however, their primary function there seems to be the first-pass extrahepatic ethanol metabolism [30]. Thus, the oxidation rather than reduction appears to be the main reason of the aldehydes’ uptake noted within this study [30].

The decrease of *n*-butyl acetate can mirror the activity of carboxylesterases (CESs), enzymes ubiquitous in human tissues [31]. This ester could be hydrolysed by CESs into acetic acid and 1-butanol being subsequently converted into *n*-butanal by ADHs, and next butanoic acid by ALDHs.

The uptake of aldehydes has already been noted in human cells cultures. Filipiak et al. [14, 15] and Sponring et al. [12] reported the consumption of species from this chemical family by lung cancer and normal cells. In our recent paper [16] we evidenced similar phenomenon in cultures of human hepatocellular carcinoma cells (HepG2). ALDHs are particularly expressed in both lung and liver cells [27], moreover, their activity is additionally increased in their cancer counterparts [32, 33]. Both lung and liver cells were also shown to metabolise *n*-butyl acetate during in vitro studies [12, 14–16].

### Emission of VOCs by HUVEC

Eight compounds increased their levels at the presence of HUVEC (see Table 3). Amongst them there were three esters (ethyl acetate, ethyl propanoate and ethyl butyrate), three ketones (3-heptanone, 2-octanone and 2-nonanone), one volatile sulphur compound (2-methyl-5-(methylthio) furan) and one aromatic hydrocarbon (toluene). The highest concentrations were observed for ethyl acetate (mean of 10.1 ppb in cell cultures vs. 1.8 ppb in media) and toluene (7.8 vs. 3.6, respectively). However, the toluene levels increased only by a factor of two, whereas the ethyl acetate ones almost six-fold.

Ketones production by the HUVEC can be attributed to the aforementioned high expression of alcohol dehydrogenases (ADHs) in human vascular endothelium [30]. Although primary alcohols seem to be the most preferred substrates for ADHs, they can also oxidise longer-chain cyclic and secondary alcohols [27, 34–36]. The latter were shown to be rather poor substrates for ADHs [36], nevertheless their conversion into ketones has been documented in the literature [34, 36]. Consequently, 2-octanone could be the product of the 2-octanol oxidation and 2-nonanone possibly stemmed from 2-nonanol. The origin of these secondary alcohols remains unclear. Probably they were present in small amounts in the applied medium. An alternative pathway leading to the formation of ketones in humans employs β-oxidation of branched-chain fatty acids. For example, valproic acid was demonstrated to be metabolised into 3-heptanone [37] and 2-ethylhexanoic acid was reported to be oxidised to 2-heptanone and 4-heptanone [38]. The respective branched-chain fatty acids can in turn be the metabolites of the appropriate branched-chain primary alcohols or/and aldehydes (e.g. 2-propyl pentanol or 2-propyl pentanal in case of 3-heptanone). However, it is not clear if these substrates were present in the applied medium.

Interestingly, all esters found to be released by HUVEC stemmed from ethanol. Indeed, huge amounts of this alcohol (exceeding the dynamic range of the MS detector) were detected in the head-space of both cell cultures and blanks. Consequently, it seems plausible that the esterification reaction involving ethanol and the respective fatty acids could induce the production of the observed esters. Although such a reaction in the absence of a catalyst is very slow and the products relatively unstable, small amounts of esters could form and go into the gas phase. Thus, ethyl acetate was presumably generated by a reaction of ethanol with acetic acid—a product of the oxidation of the former by a tandem of ADHs and ALDHs. The high concentrations of ethyl acetate as compared with the other liberated esters seem to confirm this hypothesis. Analogously, propanoic and butanoic acids—substrates necessary for the production of the remaining esters—could in turn be produced from 1-butanol and 1-propanol (or *n*-propanal and *n*-butanal). Apart from 1-butanol, all these potential substrates were found in the head-space of the cell cultures. Consequently, the release of esters seems to be an indirect reflection of ADH and ALDH activities.

The origin of toluene and 2-methyl-5-(methylthio)-furan remains unclear; however, the latter was found to be produced also by human hepatocellular carcinoma cells [16].

The release of ketones was reported also in case of lung and liver cells [13, 15, 16], which is consistent with the ADHs’ expression in different human tissues [27]. In terms of esters, both cancer liver and lung cells were evidenced to emit *n*-propyl acetate [15, 16].

## Conclusions

In the present study, gas chromatography with mass spectrometric detection coupled with head-space needle trap extraction (HS-NTE) as the pre-concentration technique was applied for the identification and quantification of volatiles being released or metabolised by human umbilical vein endothelial cells (HUVEC). Seventeen VOCs were found to change their levels in the presence of HUVEC cells (Wilcoxon signed-rank test, *p* < .05). Amongst the consumed species, there were eight aldehydes (2-methyl 2-propenal, 2-methyl propanal, 2-methyl butanal, 3-methyl butanal, *n*-hexanal, benzaldehyde, *n*-octanal and *n*-nonanal) and *n*-butyl acetate. Eight compounds were emitted by the cells under study. This group embraces three esters (ethyl acetate, ethyl propanoate and ethyl butyrate), three ketones (3-heptanone, 2-octanone and 2-nonanone), one volatile sulphur compound (2-methyl-5-(methylthio) furan) and one aromatic hydrocarbon (toluene). The uptake and release of majority of these analytes can be attributed to the expression of enzymes in the endothelial cells, such as ADHs and ALDHs. Thus, the analysis of volatiles released or emitted by cell lines has a potential for the identification and assessment of enzymes activities.

The uptake of aldehydes by HUVEC is particularly interesting as some species from this chemical class (e.g. *n*-pentanal, *n*-hexanal, *n*-heptanal and *n*-octanal) have been proposed as blood and breath biomarkers of various forms of cancer [28, 39–42] or oxidative stress [43, 44]. The human vascular endothelium exhibits enormous surface of 500–700 m^2^ [30]. Consequently, it is likely that blood vessels reduce significantly the blood levels of aldehydes in general and disease-related aldehydes in particular. This observation questions aldehydes as reliable markers providing the information on disease processes, or metabolic disorders occurring in distant parts of the body. Moreover, in the context of breath gas analysis, the vascular system cannot be considered as an inert trunk line transporting volatile markers to lungs. Thus, the selection of new breath markers should embrace the studies on their stability in blood and vascular system.
